# Post discharge management of heart failure patients: clinical findings at the first medical visit in a single-center study

**DOI:** 10.1186/s12872-023-03113-1

**Published:** 2023-02-20

**Authors:** F. Sall, A. Adoubi, C. Boka, N. Koffi, P. Ouattara, A. Dakoi, J. B. Anzouan-Kacou

**Affiliations:** 1 Université Alassane Ouattara, Bouake, Côte d’Ivoire; 2 Université Félix Houphouët-Boigny, Abidjan, Côte d’Ivoire

**Keywords:** Heart failure, Postdischarge, Management

## Abstract

**Background:**

The Post Discharge Management of patients with heart failure impact significantly their incomes. This study aims to analyze the clinical findings and management at the first medical visit of these patients in our context.

**Material and methods:**

This is a retrospective cross-sectional descriptive study on consecutive files of patients hospitalized for heart failure from January to December 2018 in our Department. We analyse data from the first post discharge medical visit including medical visit time, clinical conditions and management.

**Results:**

Three hundred and eight patients (mean age: 53.4 ± 17.0 years, 60% males) were hospitalized on median duration of 4 days [1–22 days]. One hundred and fifty-three patients (49,67%) were presented at the first medical visit after 66.53 days[0.06–369] on average, 10 (3.24%) patients died before this first medical visit and 145 (47.07%) had been lost to follow-up. The re-hospitalization and treatment non-compliance rates were 9.4% and 3.6%, respectively. Male gender (*p* = 0.048), renal failure (*p* = 0.010), and Vitamin K antagonist (VKA) /direct oral anticoagulant (DOAC) (*p* = 0.049) were the main lost to follow-up factors in univariate analysis without statistic signification in multivariate analysis. Hyponatremia (OR = 2.339; CI 95% = 0.908–6.027;* p* = 0.020) and atrial fibrillation (OR = 2.673; CI 95% = 1.321–5.408;* p* = 0.012) were the major mortality factors.

**Conclusion:**

The management of patients with heart failure after discharge from hospital seems to be insufficient and inadequate. A specialized unit is required to optimize this management.

## Introduction

The transition period from the start of hospitalization of patients with heart failure to a period around discharge from hospital and up to 6 months after is called the "vulnerable phase", during which the patient is at high risk of adverse events due to multiple cardiac and non-cardiac factors [1, 2] with post-hospital mortality rates of up to 15% and readmission rates of 20% to 30% in the first 30 days after discharge from hospital [3]. Thereby, the post-hospitalization management of patients with heart failure is crucial and raises 4 issues: 1- the time of the first post-hospitalization consultation, 2- the patient's clinical condition at this first consultation, 3- the post-hospitalization therapeutic optimization, 4- improved prognosis with the issue of mortality and re-hospitalizations. According to the literature [4, 5], less than a third of patients hospitalized for heart failure saw a cardiologist within the first 3 months after discharge from hospital. Thus, in the United Kingdom for example, only 56% of hospitalized patients had an organized follow-up [6]. This poor coordination is one of the major reasons why the titration of essential drugs remains suboptimal and has a considerable impact on the prognosis of patients such that approximately 1 in 4 patients is readmitted within 30 days of hospitalization and almost half within 6 months during this period called "vulnerable phase"; and 5% may die during this period [3, 7]. The most recent international guidelines recommend a follow-up visit within 7–14 days and/or a telephone follow-up within 3 days of hospital discharge [6, 8, 9]. However, data from several countries suggest that such early follow-up is exceptional [6]. Patients who overcome this period successfully can make the transition to long-term stability [1].

This study aims to analyze the management and outcomes of these patients immediately after hospitalization in our context.

## Material and methods

This is a cross-sectional retrospective study covering all consecutive files of patients hospitalized in our Department between January 1st, 2018 and December 31st, 2018. The main diagnosis retained at the discharge was heart failure according to Framingham clinical criteria. An update of the data by telephone call including the evaluation of the prognosis of the patients was carried out from January 2019 to May 05, 2019. Were included in our study, all adult patients, hospitalized for heart failure, released alive from the hospital and whose clinical course was favorable. We analyzed the data from the first post-hospitalization consultation, namely the epidemiological, clinical, therapeutic and evolutionary aspects of these patients at this first consultation. The data was collected on an individual survey sheet, filled in according to the various parameters studied, based on the medical file. Statistical analysis was carried out using Epi info 7 software and using SPPS version 27 (SPSS Inc.,Chicago,IL,USA). The qualitative values ​​were expressed as a percentage and the quantitative variables as the mean ± standard deviation. Comparisons were made with ANOVA for quantitative variables and the Chi2 test for qualitative variables. We performed a logistic regression analysis to search factors associated with the lost to follow-up and the mortality. For the logistic regression procedures, we chose relevant variables based on data from the literature [10–16], and based on our clinical practice. A p-value < 0.05 was considered statistically significant.

## Results

### Epidemiology and clinical

**Fig. 1:**
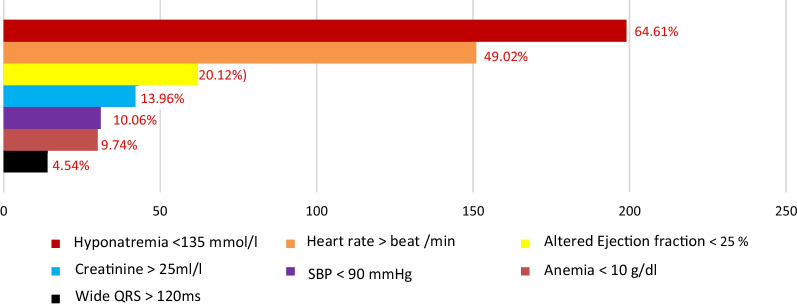
Prevalence of poor prognosis factors, SBP: systolic blood pressure

We collected 308 files of patients (mean age: 53.4 ± 17.0 years [range: 19—85 years]; 60% males).). The 60–69 age group was the largest. The clinical characteristics of the patients are listed in Table 1. Almost 52.92% of patients were at least at their 2nd episode of decompensation. Chronic decompensation (51.8%), Acute Lung Edema (28%) and low cardiac output (13%) were the most frequent clinical forms. Dilated cardiomyopathies (56.5%), valve disease (15.4%) and arterial hypertension (9.4%) constituted more than two-thirds of the causes of heart failure. Decompensations factors were dominated by bronchopulmonary infections and poor adherence to therapy, respectively at 48.7% and 40.3%. The factors of poor prognosis (morbidity and mortality) were marked by hyponatremia (64.61%), tachycardia (49.02%) and LVEF < 25% (20.12%) as shown in Fig. 1. Heart failure with altered ejection fraction constituted 77% of the total. The evolutionary and therapeutic data were variable as shown in Table 2. The median length of hospitalization was 4 days [1–22 days]. Exit therapy consisted of diuretics in 93.2% of cases, beta blockers in 70.1% of cases and ACE inhibitors in 63.6% of cases.

**Table 1:** Characteristics of patients hospitalized for heart failure

Characteristics	N = 308
Age (years): mean age ± standard deviation	53.4 ± 17.0
Gender	
Male (n; %)	185 (60)
Average weight (kg) ± standard deviation	63.7 ± 21.6
Average height (m) ± standard deviation	1.7300 ± 0.10374
Average BMI (kg/m^2^) ± standard deviation	29.556 ± 9.45
Type of heart failure; n (%)	N = 308
Right	48 (15.6)
Left	91 (29.6)
Global	169 (54.8)
Nth episode of decompensations; n (%)*	N = 308
1st episode	145 (47.08)
2nd episode	115 (37.33)
3rd episode	31 (10.06)
4th episode	9 (3.00)
5th episode	4 (1.30)
6th episode	3 (1.00)
8th episode	1 (0.32)
Forms of heart failure; n (%)	
Chronic decompensation	100 (51.8)
Acute Lung Edema	54 (28)
Low cardiac output	25 (13)
Hyperflow	6 (3.1)
Acute right heart failure	4 (2.1)
Others	4 (2.1)
Cardiogenic shock	0
Etiologies; n (%)	N = 308
Dilated cardiomyopathy	174 (56.5)
Valvulopathy	48 (15.4)
Hypertention	29 (9.4)
Ischemic cardiomyopathy	27 (8.8)
Others	20 (6.5)
Chronic decompensation	5 (1.6)
Hypertrophic cardiomyopathy	4 (1.3)
Myocarditis	1 (0.3)
Aortic dissection	0
Severe rhythm disturbance	0
Tamponade	0
Decompensation factors; n (%)	
Bronchopulmonary infections	150 (48.7)
Poor observance	124 (40.3)
Others	88 (28.57)
Hydro soda overload	81 (26.3)
Renal dysfunction	76 (24.7)
Atrial fibrillation	54 (17.5)
Anemia	51 (16.6)
Pulmonary embolism	22 (7.1)
Ischemic	12 (3.9)
Bradycardia	3 (1)
Surgery	2 (0.6)
Alcohol and drugs	2 (0.6)
Asthma	2 (0.6)
Hyperthyroidism	1 (0.3)
Infectious endocarditis	1 (0.3)
Electrocardiographic aspects; n (%)	
Sinus rhythm	223 (72.4)
Left ventricular hypertrophy	104 (33.8)
Atrial fibrillation	85 (27.6)
Ischemia	57 (18.5)
Echocardiographic aspects; n (%)	
Altered Ejection fraction	237 (77)
Preserved Ejection fraction	71 (23)

BMI: body mass index; *Nth episode of heart failure presented by patient at current admission

**Table 2:** Hospital stay and treatment of patients hospitalized for heart failure

Characteristics	Values
Average length of hospital stay ± standard deviation (days)	6 ± 5
Exit processing; n (%)	
Loop diuretics (Furosemide)	283 (93.2)
Strong diuretics (Furosemide ≥ 250 mg)	25 (8.1)
Nitrogen derivatives	7 (2.3)
Converting enzyme inhibitors	196 (63.6)
Central antihypertensives	4 (1.2)
Renin-Angiotensin System Inhibitors	25 (8.1)
B-blocker	216 (70.1)
Ivabradine	1 (3.2)
Calcium channel blockers	39 (12.7)
Aspirin	54 (17.6)
Spironolactone	147 (47.7)
Digoxin	18 (5.8)
Anti-Vitamin K	64 (20.8)
Favorable evolution	308 (100)

**Tableau 3:** Characteristics of patients at the 1st post-hospitalization consultation

Characteristics	Values
Patient status at the first consultation	n (%)
Lost to follow-up	145 (47.07)
Died	10 (3.24)
Seen in 1st consultation	153 (49.67)
Average consultation time (days) [extremes]	66.53 [0.06–369]
Hospital readmission	29 (9.4)
Clinical evolution at the 1st consultation	N = 153
Favorable	136 (88.9)
Stationary	9 (5.9)
Unfavorable	8 (5.2)
Clinical state	N = 153
Normal	101 (66.01)
Global heart failure	22 (14.4)
Chronic decompensation	11 (7.2)
Left heart failure	8 (5.2)
Right heart failure	6 (3.9)
Low cardiac output	3 (2.0)
Acute lung edema	2 (1.3)
Hyperflow	0
Cardiogenic shock	0

### Becoming patients after hospitalization

**Fig. 2:**
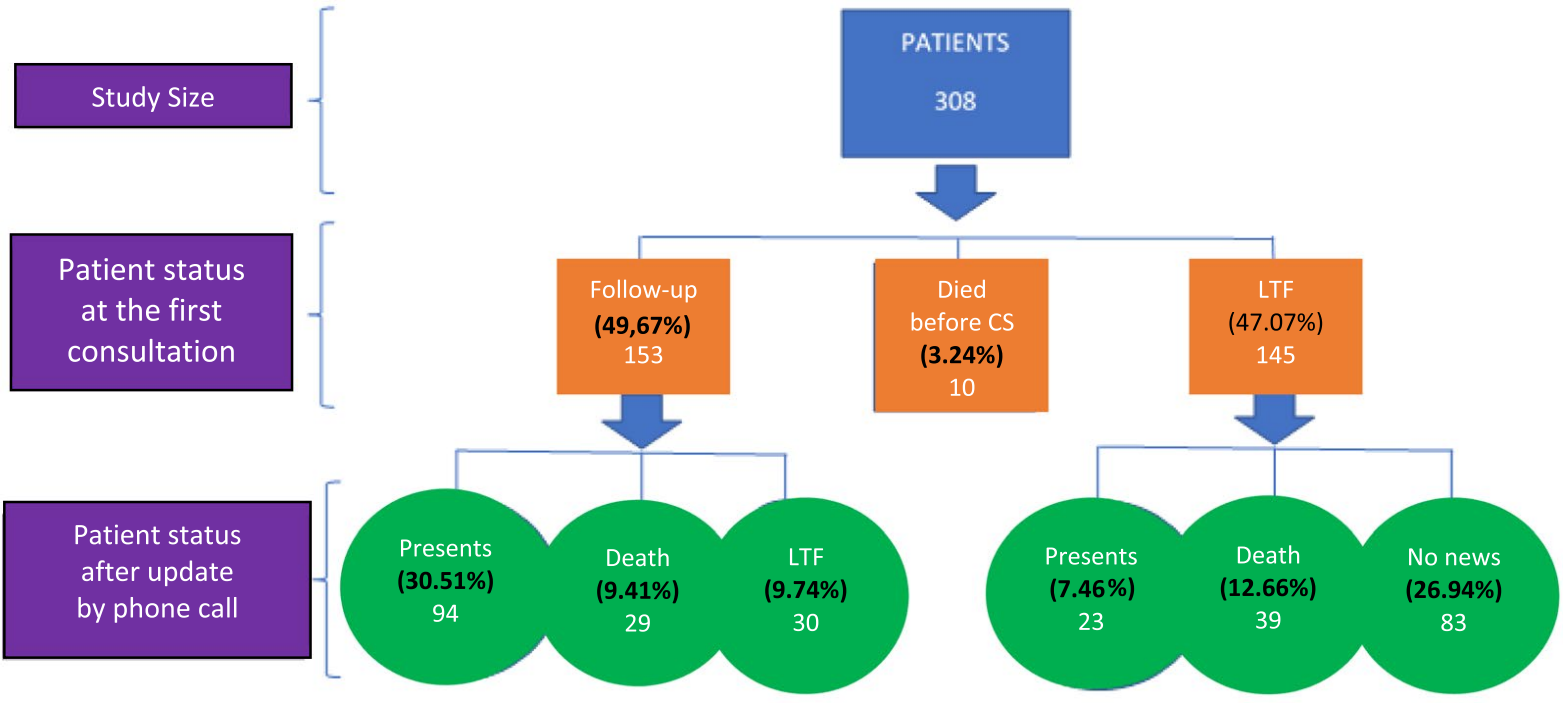
Status of patients after discharge from hospital. CS: consultation, LTF: lost to follow-up

After leaving the hospital, the patients were seen for the 1st consultation. Their post-hospitalization status was assessed by analyzing their medical records and then updated by phone call. One hundred and fifty-three patients (49.67%) presented for the first post-hospitalization consultation. There were 10 deaths before the 1st consultation (3.24%) and 145 patients lost to follow-up at the 1st consultation (47.07%) as shown in Fig. 2.

### Number of consultations

During the study period, 65 patients (21.1%) had an average of 1 consultation, 51 patients (16.5%) had an average of 2 consultations, 17 patients (5.5%) had an average of 3 consultations, 7 patients (2.3%) had an average of 4 consultations, 4 patients (1.3%) had an average of 5 consultations, 1 patient (0.3%) had an average of 6 consultations.

### Patients seen at the 1st consultation

Of 308 patients, 153 (49.7%) were seen at the 1st consultation. Regarding these patients followed, for a median expected time of 14 days, they were effectively reviewed after 66.53 days [0.06–369] on average. Of these 153 patients, 29 (9.4%) had transited to the cardiological emergency department for rehospitalization (Table 3).

### Treatment

**Fig. 3:**
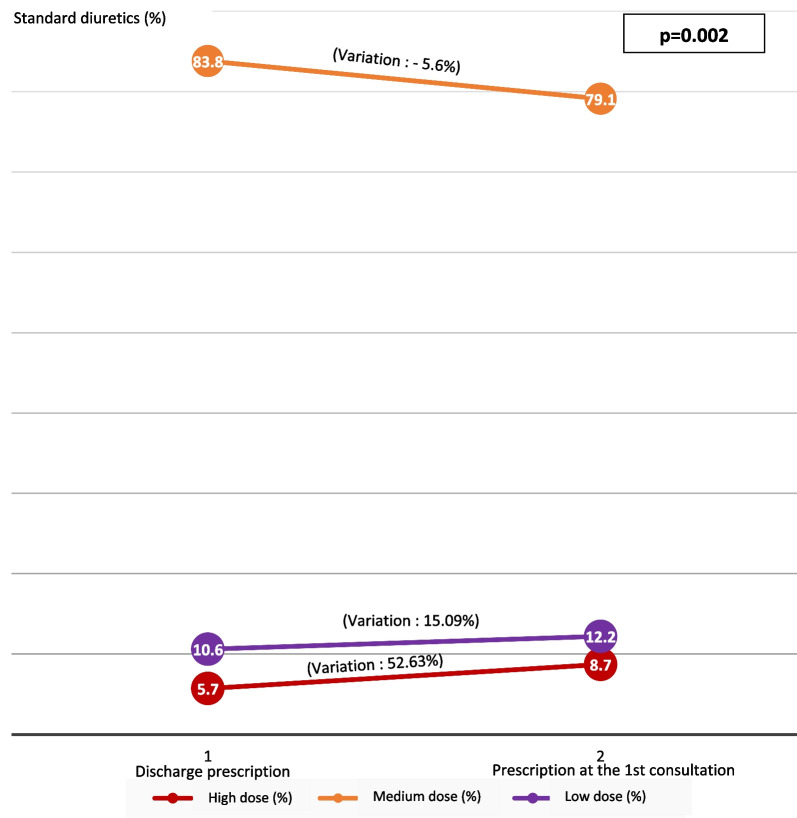
Variation in the prescription of diuretics after hospitalization

**Fig. 4:**
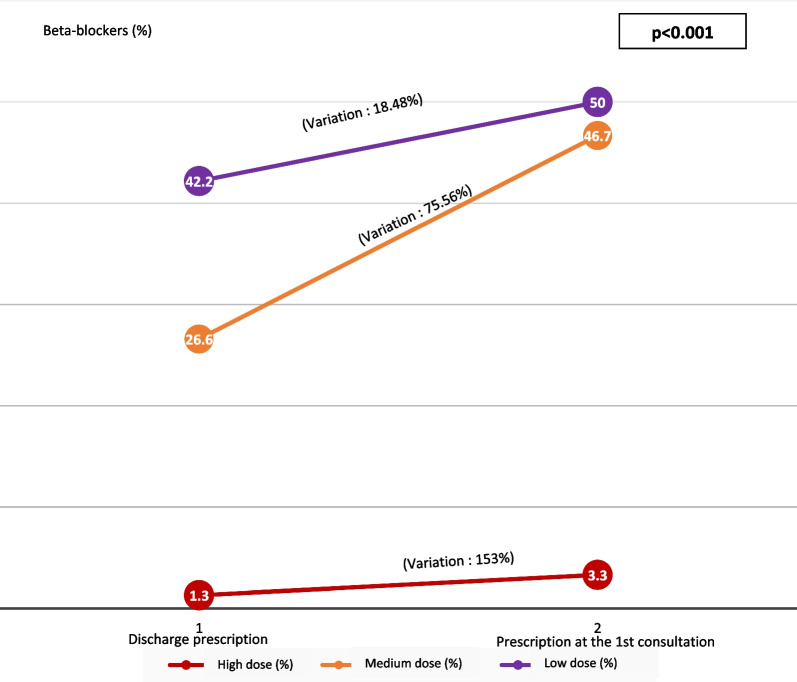
Variation in the prescription of beta-blockers after hospitalization

**Fig. 5:**
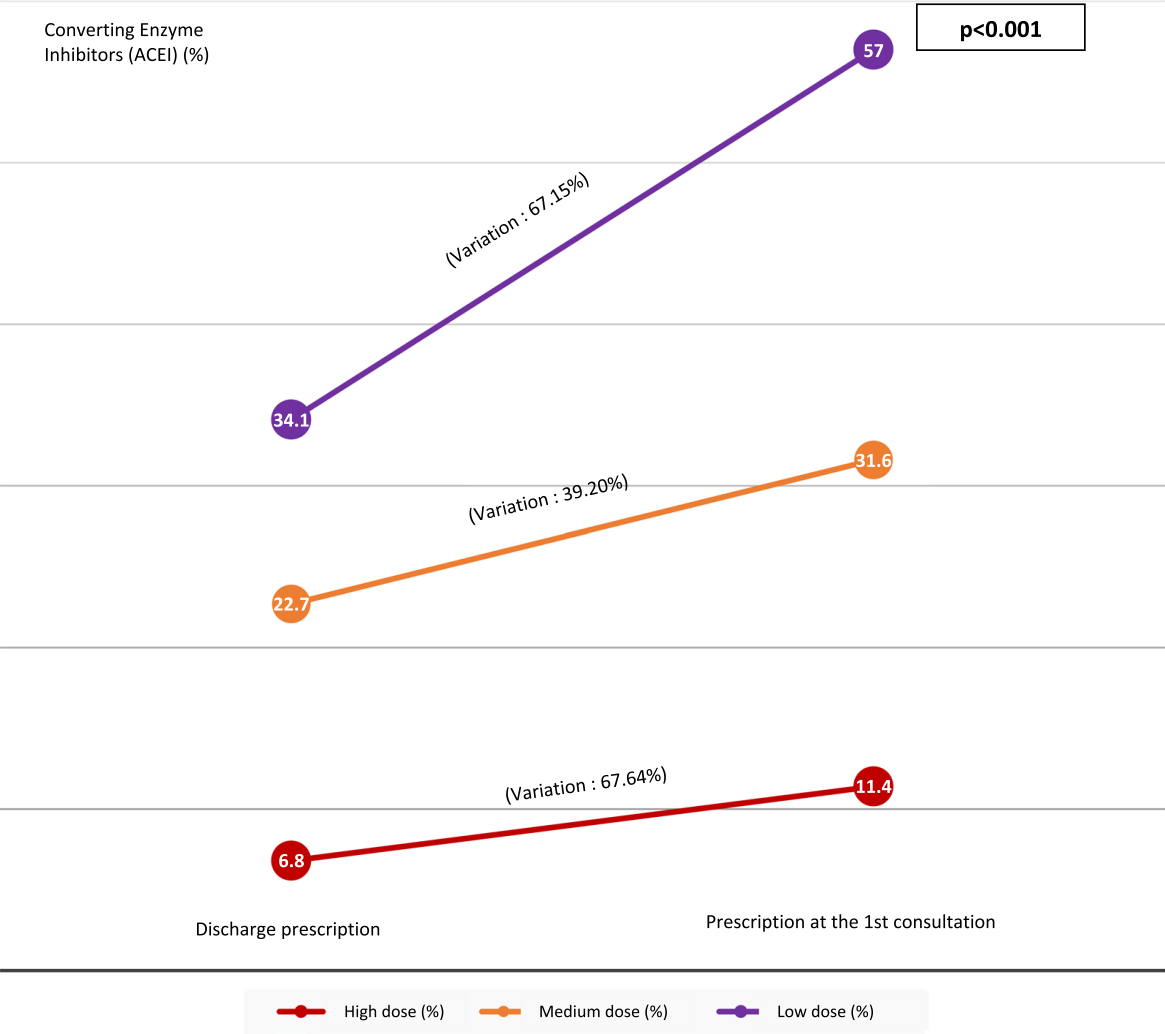
Variation in the prescription of converting enzyme inhibitors (ACEI) in post-hospitalization

At this 1st consultation, the treatment of heart failure was increased; including doses of diuretics, beta blockers and ACE inhibitors (Figs. 3, 4, 5). Were considered as low dose, medium dose and high dose of diuretics (Furosemide), respectively, the doses of diuretics (Furosemide) less than 80 mg/day, between 80 and 120 mg/day and between 120 and 250 mg/day. The low dose, medium dose and high dose of b-blockers (Nebivolol or Bisoprolol), respectively, were doses less than 2.5 mg/day, doses of between 2.5 and 7.4 mg/day and doses between 7.5 and 10 mg/day. The low dose, medium dose and high dose of converting enzyme inhibitors (Perindopril or Ramipril), respectively, were the doses less than 2.5 mg/day, the doses between 2.5 and 7.4 mg/d and doses between 7.5 and 10 mg/d.

### Factors associated with loss of follow-up

Male gender (*p* = 0.048), renal failure (*p* = 0.010), and AOD AVK anticoagulation (*p* = 0.049) were factors associated with patients' loss of follow-up in univariate analysis (Table 4). Without statistic signification in multivariate analysis (Table 5). However, after an update of the data by phone call, it turned out that ten patients (3.2%) died before the first consultation.

**Table 4:** Factors associated with the “loss of follow-up in patients with heart failure

Variables	Follow-up n = 163	Lost of follow-up n = 145	*p *value
Gender			
M	90 (48.9%)	94 (51.1%)	0.048
Origin			
Abidjan	105 (75.5%)	102 (76.7%)	0.468
Nationality			
Ivorian	135 (82.8%)	120 (82.8%)	0.553
1st episode of decompensation			
Y	63 (38.7%)	65 (44.8%)	0.263
Atrial fibrillation			
Y	50 (30.7%)	35 (24.1%)	0.124
Renal failure			
Y	31 (19.0%)	45 (31.0%)	0.01
Anemia			
Y	26 (16.0%)	25 (17.2%)	0.439
AVK-AOD anticoagulation			
Y	40 (24.5%)	24 (16.6%)	0.049

Y: Yes; M: Male

**Table 5:** Multivariate logistic regression analysis of the “loss of follow-up” risk factors for patients with heart failure

Variables	Odds ratio	95% CI	*p *value
Gender			
M	1.492	0.920–2.419	0.105
Origin			
Abidjan	0.69	0.390–1.22	0.202
Nationality			
Ivorian	0.836	0.413–1.692	0.619
Atrial fibrillation			
Y	0.923	0.489–1.743	0.805
Renal failure			
Y	1.628	0.912–2.907	0.099
Anemia			
Y	1.38	0.578–3.296	0.468
AVK-AOD anticoagulation			
Y	0.974	0.579–1.638	0.92

Y: Yes; M: Male

### Factors associated with mortality

Hyponatremia (OR = 2.339; CI 95% = 0.908–6.027;* p* = 0.020) and atrial fibrillation (OR = 2.673; CI 95% = 1.321–5.408;* p* = 0.012) were the major factors of poor prognosis (Table 6).

**Table 6:** Multivariate logistic regression analysis of mortality risk factors for patients with heart failure

Variables	Odds ratio	95% CI	*p *value
Age	1.001	0.984–1.018	0.951
Gender			
M	0.889	0.489–1.616	0.70
Right heart failure			
Y	1.616	0.701–3.724	0.260
Left heart failure			
Y	0.781	0.352–1.731	0.542
Atrial fibrillation			
Y	2.673	1.321–5.408	0.012
Renal failure			
Y	1.140	0.567–2.293	0.713
Anemia			
Y	2.339	0.908–6.027	0.078
Hyponatremia			
Y	2.129	1.128–4.019	0.020
Length of stay	0.991	0.942–1.042	0.715

Y: Yes; M: Male

## Discussion

International guidelines recommend that patients hospitalized for heart failure undergo a clinical examination by a clinician experienced in heart failure within 7 to 14 days post-hospitalization [8, 17–19]. The goal is to provide a high-quality transition to ambulatory and community care when possible. Ideally, patients should be enrolled in a structured multidisciplinary program [6]. Indeed, despite an apparent clinical and hemodynamic improvement; and due to multiple cardiac and non-cardiac factors, patients early in the post-hospitalization period often present with worsening signs and symptoms of congestion and marked deterioration in hemodynamic and renal function [3]. Some of these abnormalities have prognostic significance influencing early mortality and/or re-hospitalization. Therefore, a follow-up visit within 1 to 2 weeks is recommended [3]. This follow-up visit is an ideal opportunity to initiate or increase the titration of the medication [20].

In our series, of 308 patients hospitalized for heart failure, 153 (49.67%) were seen at the 1st post discharge consultation after 66.53 days [0.06–369] on average. This delay were abnormally high compared to international standards. One hundred and forty-five (47.07%) did not present at the first visit after hospitalizations and were lost to follow-up. Male gender (*p* = 0.048), renal failure (*p* = 0.010), and VKA AOD anticoagulation (*p* = 0.049) were the factors associated with this loss to follow-up. Ten patients (3.2%) died before the first consultation. Apart from the study by Msadek [21] conducted in 2019 in France with general practitioners which alluded to the notion of lost to follow-up as an explanatory factor for the lack of post-hospitalization therapeutic optimization for heart failure, both African and Western studies on the issue of loss of sight in patients with heart failure in the post-hospitalization period seem to be almost non-existent and it is difficult to compare our results with data from the literature.

The clinical condition of patients seen at the first post discharge consultation was favorable in 86.8%, stationary in 7% and unfavorable in 6.3% with re-hospitalization in 9.4% of cases. At this 1st post-hospitalization consultation, the treatment of heart failure was increased, in particular the doses of diuretics, converting enzyme inhibitors and beta blockers as recommended by learned societies [22, 23].

The rate of therapeutic non-compliance at the 1st consultation was estimated at 3.6%. This rate of therapeutic non-compliance is comparable to that of 5.8% reported by Chioncel [2] in Romania in 2018. However, it is lower than those of Ambrosy [24] in 2014 and Jackevicius [25] in 2015 both in the USA who found 8.9% and 30% respectively. According to the World Health Organization (WHO), there are 5 categories multifactorial causes of the therapeutic non-compliance: socioeconomic factors, factors associated with the health care team and system in place, disease-related factors, therapy-related factors, and patient-related factors [26]. Furthermore, the WHO supports that increasing the effectiveness of adherence interventions can have a much greater impact on the health of the population than any improvement in specific medical treatments [27]. Specifically, for patients with HF, several studies have shown that medication nonadherence was associated with an increased risk of mortality and readmissions [28–31]. Complex and independent factors affect treatment adherence. According to the WHO, these are factors linked to the health system, to the disease itself and its treatment, to the socio-economic status, and the level of education of the patient, to the patient-provider relationship, the fluctuating nature of HF, the acute and chronic nature of HF, and the patient's knowledge of their disease [32, 33]. Among the current interventions proposed to improve patient compliance after discharge from hospital, the one that appears to be the most relevant and effective is the initiation of medical treatment for heart failure in hospital [34, 35].

### Perspectives

These findings highlight the need for the training of a specialized and multidisciplinary unit to optimize the treatment of patients with cardiac insufficiency after discharge from the hospital, and the promotion of tele-consultation to improve the follow-up of these patients. This is a preliminary study that requires further, more in-depth studies and which, despite its limitations linked to the retrospective and monocentric nature, the large number of lost to follow-up retains all its originality.

### Limitations

The limits of this study are linked to the retrospective and monocentric nature and the large number of lost to follow-up.

## Conclusion

At the end of this study, our observation is that the time taken for the first consultation of these patients after discharge from the hospital is abnormally long the management seems to be insufficient and inadequate. Therefore, it is important to recognize that the management of this chronic condition follows a continuum, and that post-hospital treatment and follow-up are as important as hospital care. Better overall organization of medical care centered on patients with heart failure is therefore essential and requires a specialized unit to optimize treatment. Thus, a pharmaco-economic evaluation of new initiatives would be carried out to select the optimal strategies. In addition, seeing these patients again, preferably before 2 weeks after hospitalization, could help avoid a significant risk of death.

## Data Availability

The datasets used and/or analysed during the current study available from the corresponding author on reasonable request.
